# TEG6s Platelet Mapping assay for the estimation of plasma fibrinogen concentration during cardiovascular surgery: a single-center prospective observational study

**DOI:** 10.1007/s00540-021-03009-4

**Published:** 2021-10-13

**Authors:** Yudai Yamamoto, Yunosuke Sato, Miri Takahashi, Hiroto Yamamoto, Mayumi Echizen, Tokujiro Uchida

**Affiliations:** 1 Department of Anesthesiology, Graduate School of Medical and Dental Sciences, Tokyo Medical and Dental University, 1-5-45 Yushima, Bunkyo-ku, Tokyo, 113-8510 Japan; 2 Department of Anesthesiology, Medical Hospital, Tokyo Medical and Dental University, Tokyo, Japan

**Keywords:** Cardiopulmonary bypass, Fibrinogen, Platelet Mapping, Activator F, Thromboelastography

## Abstract

**Purpose:**

The Activator F (ActF) test on the TEG6s Platelet Mapping assay system is a means of quantifying blood viscoelasticity caused by fibrin network formation, triggered by reptilase and factor XIII, while platelets are inhibited. This unique methodology enables the measurement of blood viscoelasticity, even in highly heparinized blood. Here, we investigated whether fibrinogen concentration could be estimated using the ActF test in blood samples obtained during cardiopulmonary bypass (CPB) and after CPB in patients undergoing cardiovascular surgery.

**Methods:**

We performed a single-center prospective observational study at a university hospital. Forty patients aged ≥ 18 years who underwent elective cardiovascular surgery with CPB were enrolled. Blood samples were drawn after the induction of anesthesia, after declamping of the aorta during CPB, and after the reversal of heparinization using protamine (after CPB). Coagulation profiles were evaluated using the Platelet Mapping assay and standard laboratory tests.

**Results:**

There were strong correlations between the maximal amplitude of clot strength (MA) in the ActF test and fibrinogen concentration in samples drawn during CPB (*R* = 0.84, 95% confidence interval [CI] 0.72–0.91; *P* < 0.001) and after CPB (*R* = 0.83, 95% CI 0.70–0.91; *P* < 0.001). The areas under the receiver-operating characteristic curve for the ActF MA for fibrinogen concentrations < 150 mg/dL were 0.86 (95% CI 0.73–1.0) during CPB and 0.98 (95% CI 0.94–1.0) after CPB.

**Conclusion:**

TEG6s Platelet Mapping ActF MA values strongly correlated with plasma fibrinogen concentration in highly heparinized blood during CPB and yielded highly accurate measurements of low fibrinogen concentrations.

**Supplementary Information:**

The online version contains supplementary material available at 10.1007/s00540-021-03009-4.

## Introduction

Cardiovascular surgery with cardiopulmonary bypass (CPB) often results in a coagulopathy that is caused by hemodilution, the consumption of clotting factors, altered platelet function, and hypothermia [1–3]. Coagulation management using viscoelastic hemostatic assays has been shown to reduce the volume of blood requiring transfusion during cardiovascular surgery and is recommended in published guidelines [4–8]. Fibrinogen is the most rapidly depleted clotting factor during massive bleeding [9]. A reduction of fibrinogen concentration increases blood transfusion volume and affects clot formation [10, 11]. Therefore, information regarding the plasma fibrinogen concentration is important during hemostatic management. However, central laboratory test methods require 30–50 min to provide information regarding the plasma fibrinogen concentration. Viscoelastic hemostatic assays provide rapid results concerning plasma fibrinogen concentrations; thus, these methods are more suitable for cardiovascular surgery, where rapid hemostatic management is necessary [12, 13]. Several transfusion strategies have been proposed with cutoff values based on the relationship between fibrinogen concentrations and viscoelastic hemostatic assay findings [14, 15]. Therefore, it is important to verify the relationship between viscoelastic hemostatic assay findings and fibrinogen concentrations.

Furthermore, the evaluation of blood coagulation during CPB is reportedly useful for the early management of blood coagulation after CPB [14, 16–19]. Some reports have shown that blood transfusion algorithms involving viscoelastic hemostatic assays during CPB reduce the amount of transfused blood, suggesting that viscoelastic hemostatic assays are clinically useful during CPB [20, 21].

The recently introduced TEG6s (Haemonetics, Braintree, MA, USA) is a device for evaluating viscoelastic coagulation that measures changes in resonance when whole-blood samples are subjected to vibration [22]. When compared with conventional blood viscoelasticity tests, the TEG6s requires a smaller blood sample (300 μL), and all the measurement processes are completed automatically. However, coagulation parameters of highly heparinized samples cannot be measured using the Global Hemostasis assay, a coagulation test performed on the TEG6s using kaolin and tissue factor as reagents [23], and it is therefore difficult to assess the blood coagulation profile of patients before heparin neutralization during cardiovascular surgery with CPB using this assay system.

TEG6s Platelet Mapping (Haemonetics) is a method for the assessment of platelet function that uses thrombin and ADP receptor stimulation. Activator F (ActF) is a component of this assay that can be used to quantify blood viscoelasticity caused by fibrin network formation triggered by reptilase and factor XIII, while platelet activity is inhibited by glycoprotein (GP) IIb/IIIa inhibitors. This unique methodology should enable the measurement of blood viscoelasticity even in highly heparinized blood samples, because it is independent of thrombin. Although the results of a small preliminary study were consistent with this hypothesis [24], the usefulness of the values provided by the ActF test as surrogates for plasma fibrinogen concentration during CPB has not been established. In the present study, we therefore measured plasma fibrinogen concentration and carried out Platelet Mapping assays during and after CPB, in addition to conducting routine Global Hemostasis assay measurements after CPB, to determine if fibrinogen concentration could be assessed using the ActF test. We also compared the maximal amplitude of clot strength (MA) yielded by the ActF test (MA_ActF_) in the Platelet Mapping assay and the functional fibrinogen test (MA_CFF_), a tissue factor activation test under GP IIb/IIIa inhibition in the Global Hemostasis assay after CPB, to clarify the difference between these parameters.

## Methods

We conducted a single-center prospective observational study between August 2019 and January 2020. The study was approved by the institutional ethics committee (M2018-203) and registered in the University Hospital Medical Information Network Clinical Trial Registry (UMIN000034731). Forty patients (≥ 18 years old) who underwent elective cardiovascular surgery with CPB were enrolled. Written informed consent was obtained from all the enrolled patients. Preoperative antiplatelet therapy was stopped at least 7 days before the surgery, warfarin was stopped at least 4 days before, and direct oral anticoagulant administration was stopped at least 2 days before, according to the published guidelines [5].

### Data collection

The baseline characteristics of the participants (age, sex, height, body mass, and preoperative coagulation test results) and perioperative data were collected from the clinical recording system.

Blood samples were collected at three time points: after the induction of anesthesia (baseline), after declamping of the aorta during CPB, and after heparin reversal using protamine (after CPB), via an arterial cannula. The samples were dispensed into 2.0-mL tubes containing 0.2 mL of 3.2% sodium citrate (Becton Dickinson, Franklin Lakes, NJ, USA) for coagulation assays, 4.0-mL tubes containing heparin lithium (Becton Dickinson) for TEG6s Platelet Mapping, and 2.0-mL tubes containing EDTA (Becton Dickinson) for complete blood cell counting using an automated counter (MEK-6500; Nihon Kohden, Tokyo, Japan). Citrated blood samples were centrifuged immediately and platelet-poor plasma was stored at − 80 °C. Prothrombin time international normalized ratio (PT-INR), activated partial thromboplastin time (aPTT), and plasma fibrinogen concentration, determined by the Clauss method, were measured in platelet-poor plasma by a clinical laboratory testing company (SRL, Tokyo, Japan).

### Platelet Mapping assay

In addition to the routine Global Hemostasis assay measurements, the TEG6s Platelet Mapping assay was performed within 30 min of sample collection, according to the manufacturer’s instructions. Platelet Mapping cartridges were inserted into the TEG6s device and 300 µL blood samples collected in heparin-filled tubes were placed in the cartridge to start the measurement. The measurements were performed automatically by the cartridges, which contained reagents for three measurements: a kaolin and heparinase activation test (HKH), an ADP activation test, and ActF. The ActF test used abciximab (a GP IIb/IIIa inhibitor) to inhibit platelet function and measure viscoelasticity caused by fibrin network formation, triggered by reptilase and factor XIII, in the presence of heparin. We recorded the MA and designated the MA value yielded by the ActF test as MA_ActF_. A flat waveform indicated that there was no change in blood viscoelasticity, and MA was therefore defined as 0 mm. All the clinical staff in charge of each case were blinded to the results of the Platelet Mapping assay.

### TEG6s Global Hemostasis assay

We performed TEG6s Global Hemostasis assays for routine intraoperative hemostatic management after CPB, according to the manufacturer’s instructions. Citrated blood was used for the Global Hemostasis assay, unlike the Platelet Mapping assay. The Global Hemostasis assay comprised four tests: a kaolin activation test (CK), a kaolin and tissue factor activation test (CRT), a kaolin and heparinase activation test (CKH), and a CFF test.

### Management of anesthesia

The surgical procedures were performed under general anesthesia, which was managed individually by the anesthesiologist in charge of each case. All the clinical staff were blinded to the results from the TEG6s Platelet Mapping test (anesthesiologists, surgeons, intensivists, nurses, and perfusionists). Hemostatic management and the transfusion strategy were guided by the TEG6s Global Hemostasis assay results. All the participants were administered a single bolus of tranexamic acid (1000 mg), followed by a continuous infusion (2 mg/kg/h), until sternal closure.

### Management of CPB

This was an observational study and CPB management was therefore performed using the standard institutional practices. The CPB circuit included a membrane oxygenator (CAPIOX FX15/25; Terumo, Tokyo, Japan, or Quadrox-i Adult; Getinge Group Japan K.K., Tokyo, Japan) and a centrifuge pump (CAPIOX, Terumo). The circuit was primed using crystalloid solution (Physio 140 Injection; Otsuka Pharmaceutical Factory, Inc., Tokushima, Japan) (300–1000 mL), 6% hydroxyethyl starch 130/0.4 in 0.9% sodium chloride injection (Voluven 6% solution for infusion; Fresenius Kabi Japan K.K., Tokyo, Japan) (0–500 mL), cefazolin (2 g), mannitol (2 mL/kg), and unfractionated heparin (5,000 U). Nonpulsatile flow was maintained at > 2.3 L/min/m^2^ and the target mean arterial pressure was 50–70 mmHg during CPB. When the patient’s hematocrit was < 23% during CPB, transfusion of packed red blood cells and fresh frozen plasma (FFP) was performed, according to the institutional CPB management protocol. HMS Plus (Medtronic, Minneapolis, MN, USA) was used for the intraoperative management of heparin and protamine. Heparin was administered to a target concentration of 3.4 units/mL (2.5 mg/kg) and a kaolin-activated clotting time (ACT) ≥ 400 s. During CPB, additional heparin was administered to maintain an ACT of ≥ 400 s and a target heparin concentration of 3.4 U/mL. Protamine administration after CPB was based on the HMS Plus test results.

### Statistical analysis

The primary outcome of the study was the relationship between MA_ActF_ and plasma fibrinogen concentration during and after CPB.

Both Platelet Mapping ActF and Global Hemostasis CFF measure the contribution of fibrin to clot strength. However, CFF measures the formation of fibrin in response to the stimulation of thrombin by tissue factor, while ActF measures the formation of fibrin in response to reptilase. The clot strength may thus differ in each case. Because the Global Hemostasis measurements were performed after protamine administration following CPB, to aid the management of blood coagulation in each patient, we compared the MA yielded by the Global Hemostasis CFF test and the Platelet Mapping MA_ActF_ after CPB as a secondary outcome. The correlation between MA_ActF_ during CPB and fibrinogen concentration or MA_ActF_ after CPB was also compared.

Continuous data are summarized as median [25th, 75th percentile] and categorical data are summarized as frequencies. Pearson’s correlation coefficient (*R*) was used to evaluate the relationships between TEG6s parameters and laboratory blood coagulation test results. Receiver-operating characteristic (ROC) curves were constructed and the areas under the curve (AUC) were calculated to evaluate the accuracy of MA_ActF_ for the measurement of fibrinogen concentrations of 150 or 200 mg/dL [5, 10, 11].

*P* < 0.05 was considered to represent statistical significance. The following commonly used interpretations of correlation strength were used: 0.00–0.20, “negligible”; 0.20–0.39, “weak”; 0.40–0.69, “moderate”; 0.70–0.89, “strong”; and 0.90–1.0, “very strong” [25]. All statistical analyses were performed using R version 3.3.2 (https://www.R-project.org/) and EZR (Saitama Medical Center, Jichi Medical University, Saitama, Japan), which is a graphical user interface for R [26].

We estimated that the correlation coefficient for the relationship between fibrinogen and MA_ActF_ would be ≥ 0.6, on the basis of previous reports of the correlation between CFF and fibrinogen concentration [27]. Considering the accuracy of the equipment, an estimated sample size of 34 was required, to provide a power of 0.9, assuming an α of 0.01. After taking into account the likelihood of measurement failure and the inability to collect blood samples, the sample size was set at 40 patients.

## Results

Forty patients participated in the study between August 2019 and January 2020. Seventeen patients underwent valve surgery, 13 underwent aortic surgery, five underwent coronary bypass and valve surgery, three underwent minimally invasive valve surgery, and two underwent removal of an atrial tumor. The characteristics of the participants, preoperative coagulation test values, and surgical data are presented in Table 1.

**Table 1:** Demographic data

Female:male	16:24
Age (years old)	67 [53, 76]
Height (cm)	163 [154, 170]
Weight (kg)	59 [50, 73]
Hemoglobin (g/dL)	12.8 [11.8, 13.9]
Platelet (10^4^/μL)	18.8 [15.2, 23.1]
PT-INR	1.02 [0.97, 1.12]
aPTT (sec)	30.8 [28.5, 33.6]
Procedure (*n*)	
Valve(s)	17
Valve/CABG	5
Aorta	13
MICS	3
Removal of atrial tumor	2
Transfusion during CPB (units)	
PRBC	4 [0, 8]
FFP	8 [0, 16]
Duration of CPB (min)	287 [205, 342]
Cross clamp time (min)	202 [125, 270]
Duration of surgery (min)	486 [346, 616]

The data are shown as median [25th, 75th percentile]

*PT-INR* prothrombin time international normalized ratio, *aPTT* activated partial thromboplastin time, *CABG* coronary artery bypass graft, *MICS* minimally invasive cardiac surgery, *CPB* cardiopulmonary bypass, *PRBC* packed red blood cell concentrate, *FFP* fresh-frozen plasma

The results of the TEG6s Platelet Mapping and laboratory coagulation tests throughout the surgery are shown in Table 2. The fibrinogen-related parameters could be measured using the Clauss method and Platelet Mapping in all the samples, whereas all of the PT-INR and aPTT values were outside the detectable range during CPB. Global Hemostasis was measured using the TEG6s in all 40 participants after CPB; however, we excluded one case in which the timing of sampling for the Global Hemostasis assessment was inappropriate for comparison with the results of the Platelet Mapping assessment. MA_ActF_ results were obtained for 110/120 measurements within 10 min from the start of measurement. The final MA_ActF_ was < 2.5 mm in the remaining 10 cases.

**Table 2:** Results of TEG6s assays and laboratory coagulation tests (*N* = 40)

	Baseline	During CPB	After CPB
Laboratory tests			
PT-INR	1.06 [1.01, 1.10]	NA	1.35 [1.31, 1.46]
aPTT (sec)	30.2 [28.8, 31.7]	NA	40.1 [32.9, 49.5]
Fibrinogen (mg/dL)	242 [215, 277]	163 [150, 176]	181 [166, 204]
Platelet (10^4^/µL)	14.3 [12.1, 16.8]	8.0 [6.0, 10.5]	7.1 [5.6, 8.5]
Platelet Mapping			
HKH			
R (min)	6.7 [5.7, 7.5]	8.7 [7.5, 9.3]	6.9 [6.0, 7.9]
Angle (degree)	71.6 [67.9, 73.6]	64.7 [58.6, 67.8]	66.5 [62.1, 69.8]
K (min)	1.3 [1.2, 1.6]	2.1 [1.8, 2.6]	1.8 [1.7, 2.3]
MA (mm)	63.4 [60.7, 65.2]	56.0 [50.9, 59.7]	56.8 [53.0, 59.5]
ActF			
MA (mm)	9.8 [5.6, 12.9]	5.6 [3.9, 9.2]	6.8 [5.3, 9.1]
Global Hemostasis			
CFF			
MA (mm)	NA	NA	14.7 [12.0, 17.0]*

The data are shown as median [25th, 75th percentile]

*During CPB* during cardiopulmonary bypass period, *after CPB* after weaning off cardiopulmonary bypass, *aPTT* activated partial thromboplastin time, *ActF* activatorF, *HKH* kaolin with heparinase, *PT-INR* prothrombin time international normalized ratio

*N = 39

**Fig. 1:**
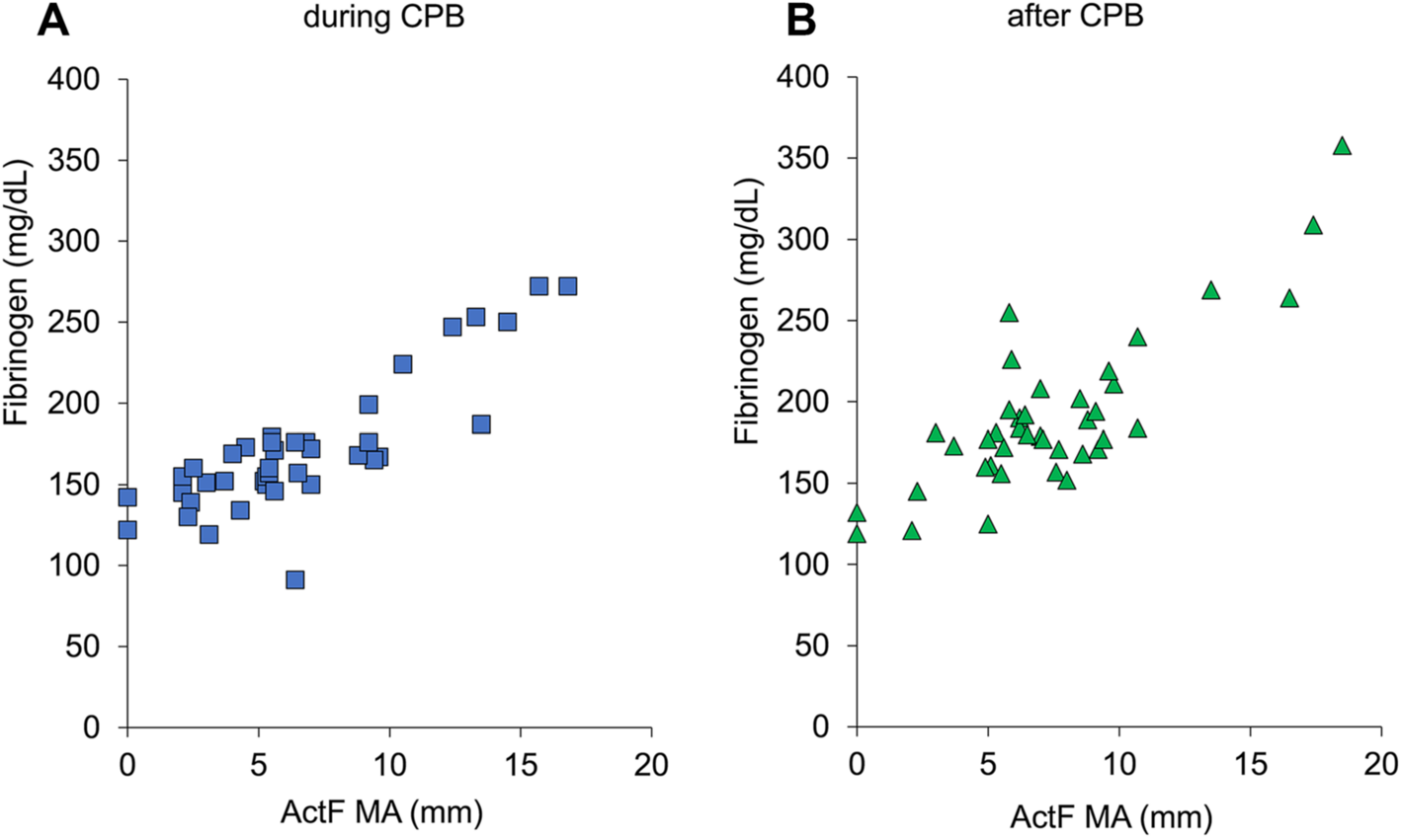
Relationship between Platelet Mapping MA_ActF_ and plasma fibrinogen concentration. **A** During CPB (*R* = 0.84, 95% CI 0.72–0.91; *P* < 0.001). **B** After CPB (*R* = 0.83, 95% CI 0.70–0.91; *P* < 0.001). During CPB: samples were drawn after the declamping of the aorta during CPB; after CPB: after heparin reversal using protamine. ActF, activator F; CI, confidence interval; CPB, cardiopulmonary bypass; MA, maximum amplitude; *R*, Pearson’s correlation coefficient

There was a strong correlation between MA_ActF_ and fibrinogen concentration in the samples drawn during CPB (*R* = 0.84, 95% confidence interval [CI] 0.72–0.91; *P* < 0.001) and after CPB (*R* = 0.83, 95% CI 0.70–0.91; *P* < 0.001) (Fig. 1).

During CPB, nine participants had fibrinogen concentrations < 150 mg/dL and 34 had concentrations < 200 mg/dL. After CPB, five patients had fibrinogen concentrations < 150 mg/dL and 29 patients had concentrations < 200 mg/dL.

**Fig. 2:**
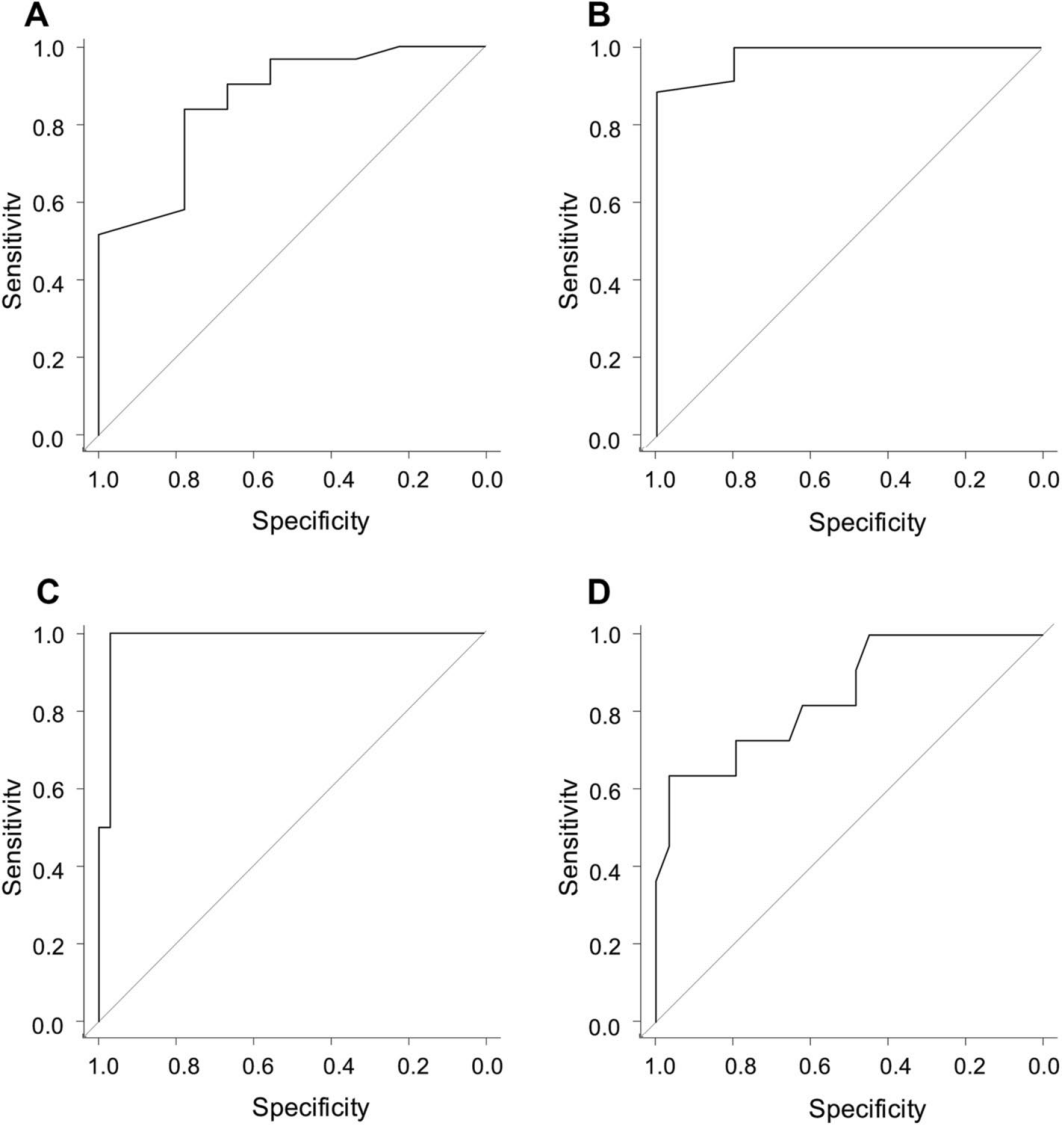
Receiver-operating characteristic curve analysis of MA_ActF_ for the measurement of fibrinogen concentrations < 150 or 200 mg/dL during and after CPB. **A** During CPB (*N* = 40, number of samples with a fibrinogen concentration < 150 mg/dL = 9). The AUC was 0.86 (95% CI 0.73–1.0). The optimal cut-off value for MA_ActF_ was 4.5 mm, with a sensitivity of 89% and specificity of 100%. **B** After CPB (*N* = 40, number of samples with a fibrinogen concentration < 150 mg/dL = 5). The AUC was 0.98 (95% CI 0.94–1.0). The optimal cutoff value for MA_ActF_ was 5.1 mm, with a sensitivity of 89% and specificity of 100%. **C** During CPB (*N* = 40, number of samples with a fibrinogen concentration < 200 mg/dL = 34). The AUC was 0.99 (95% CI 0.95–1.0). The optimal cutoff value for MA_ActF_ was 10.5 mm, with a sensitivity of 100% and specificity of 97%. **D** After CPB (N = 40, number of samples with a fibrinogen concentration < 200 mg/dL = 29). The AUC was 0.85 (95% CI 0.70–0.99). The optimal cutoff value for MA_ActF_ was 8.5 mm, with a sensitivity of 73% and specificity of 79%. AUC, area under the curve; ActF, activator F; CI, confidence interval; CPB, cardiopulmonary bypass; MA, maximum amplitude

The AUCs for the ROCs for MA_ActF_ in the measurement of fibrinogen concentrations < 150 mg/dL were 0.86 (95% CI 0.73–1.0, cutoff 4.5 mm, sensitivity 84%, specificity 78%) during CPB (Fig. 2A) and 0.98 (95% CI 0.94–1.0, cutoff 5.1 mm, sensitivity 89%, specificity 100%) after CPB (Fig. 2B). The AUCs for the ROCs for MA_ActF_ in the measurement of fibrinogen concentrations < 200 mg/dL were 0.99 (95% CI 0.95–1.0, cutoff 10.5 mm, sensitivity 100%, specificity 97%) during CPB (Fig. 2C) and 0.85 (95% CI 0.70–0.99, cutoff 8.5 mm, sensitivity 73%, specificity 79%) after CPB (Fig. 2D).

**Fig. 3:**
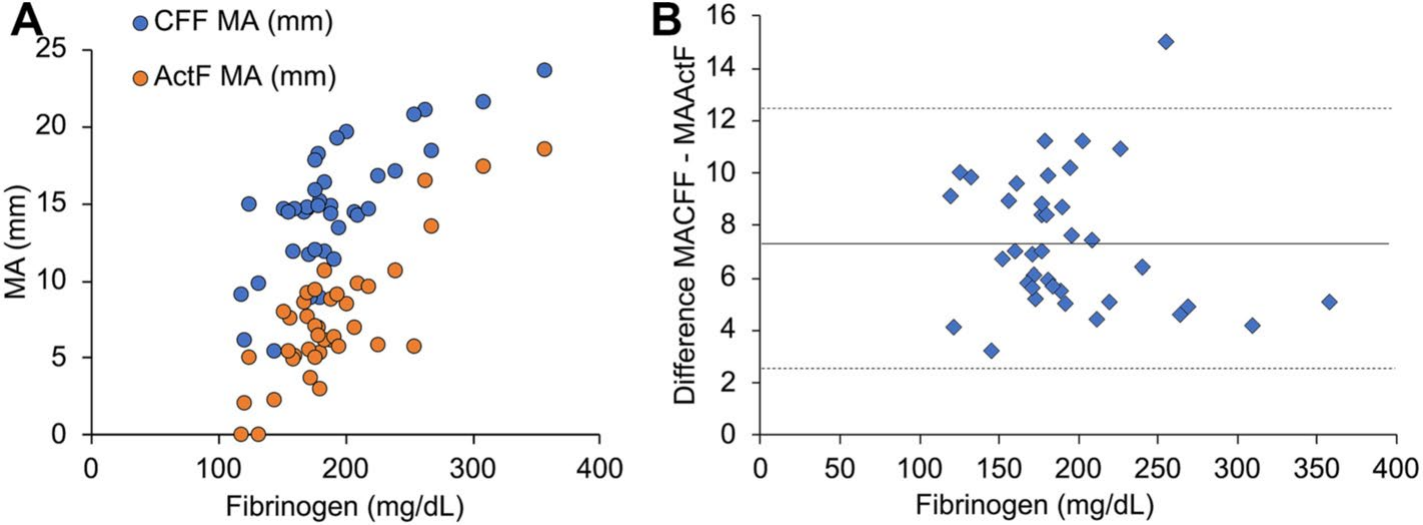
Relationships among MA_ActF_, MA_CFF_, and plasma fibrinogen concentration in samples drawn after CPB. **A** Comparison of MA_ActF_ with MA_CFF_ in the context of the relationship with plasma fibrinogen concentration. **B** Scatterplot showing the relationship between plasma fibrinogen concentration and the difference between MA_CFF_ and MA_ActF_ after CPB. The mean difference between MA_CFF_ and MA_ActF_ was 7.3 mm (95% CI 2.3–12.3 mm). After CPB, after heparin reversal using protamine; ActF, activator F; CI, confidence interval; CFF, functional fibrinogen assay as part of the Global Hemostasis assessment; CPB, cardiopulmonary bypass; MA, maximum amplitude; *R*, Pearson’s correlation coefficient

The relationships of MA_ActF_ and MA_CFF_ with plasma fibrinogen concentration are shown in Fig. 3A. MA_CFF_ was strongly correlated with fibrinogen concentration: *R* = 0.75 (95% CI 0.57–0.86; *P* < 0.001, *N* = 39). The correlation between MA_ActF_ and MA_CFF_ was *R* = 0.81 (95% CI 0.66–0.89; *P* < 0.001, *N* = 39). The relationship between plasma fibrinogen concentration and the difference between MA_CFF_ and MA_ActF_ is shown in Fig. 3B. The mean difference between MA_CFF_ and MA_ActF_ was 7.3 mm (95% CI 2.3–12.3 mm). The correlation coefficient for the relationship between plasma fibrinogen concentration and this difference was − 0.15 (95% CI − 0.44–0.18), implying that these two parameters were not significantly related.

**Fig. 4:**
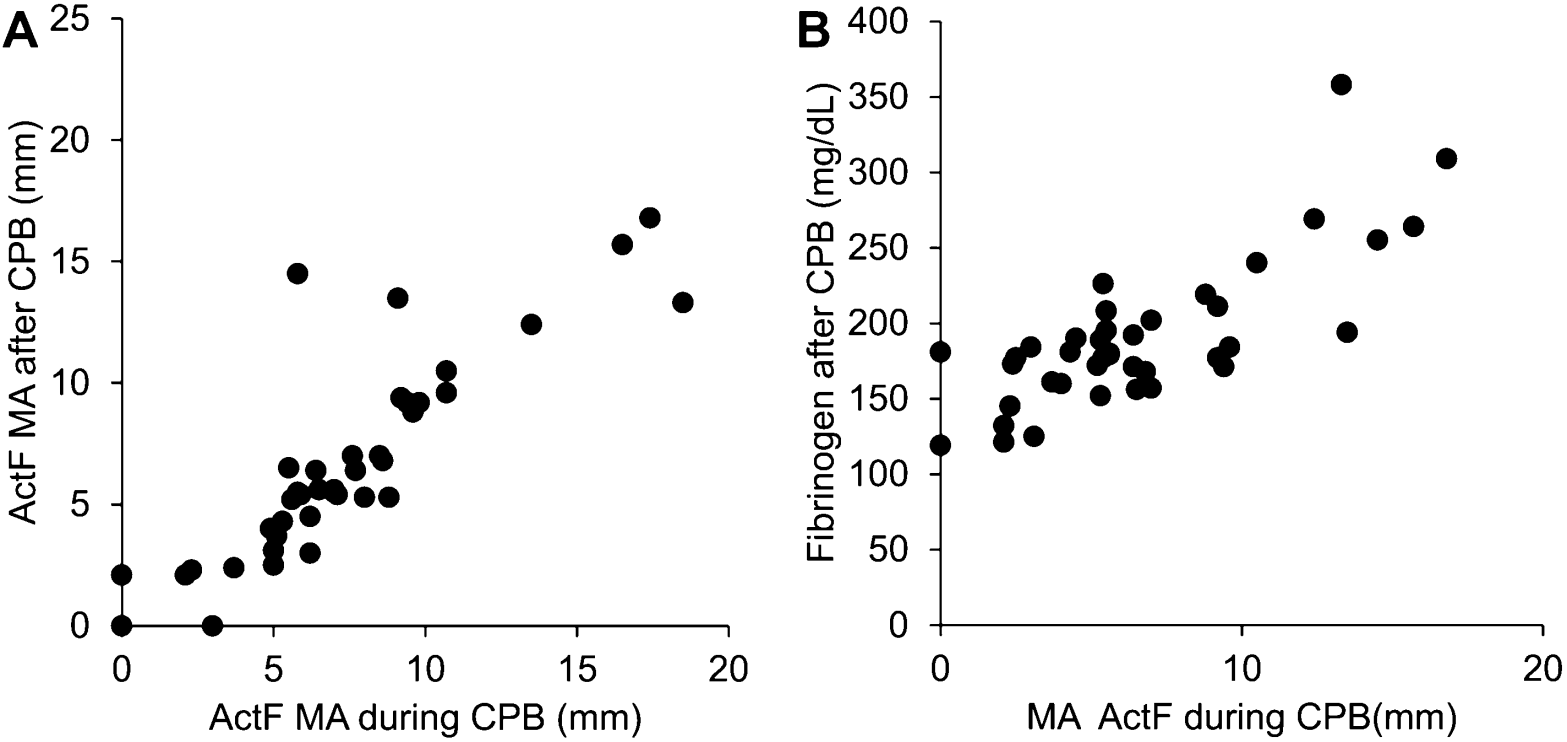
Relationships among MA_ActF_ during CPB, MA_ActF_ after CPB, and plasma fibrinogen concentration. **A** Scatter plot of MA_ActF_ during CPB and MA_ActF_ after CPB (*R* = 0.86, 95% CI 0.75–0.92; *P* < 0.001, *N* = 40). **B** Scatter plot of MA_ActF_ during CPB and plasma fibrinogen concentration after CPB (*R* = 0.78, 95% CI 0.62–0.88; *P* < 0.001, *N* = 40). During CPB: samples were drawn after the declamping of the aorta during CPB; after CPB: after heparin reversal using protamine. ActF, activator F; CI, confidence interval; CPB, cardiopulmonary bypass; MA, maximum amplitude; *R*, Pearson’s correlation coefficient

The correlation between MA_ActF_ during and after CPB was *R* = 0.86 (95% CI 0.75–0.92; *P* < 0.001, *N* = 40) (Fig. 4A) and the correlation between MA_ActF_ during CPB and the fibrinogen concentration after CPB was *R* = 0.78 (95% CI 0.62–0.88; *P* < 0.001, *N* = 40) (Fig. 4B).

## Discussion

We have shown that Platelet Mapping assays are useful for the estimation of plasma fibrinogen concentration during CPB and after protamine administration. MA_ActF_ was strongly correlated with plasma fibrinogen, even in highly heparinized samples. Erdoes et al*.* [23] previously reported that the MA_CFF_ values measured as part of the Global Hemostasis assay indicated that the blood of 91.3% patients could not clot during CPB. However, the CFF test in the Global Hemostasis assay measures thrombin-mediated blood coagulation in the absence of heparinase, and the results may therefore not have been valid in highly heparinized blood. The present results suggest that the Platelet Mapping assay may be a useful substitute for the Global Hemostasis assay, and may aid decision-making regarding the supplementation of fibrinogen before patients are weaned off CPB. The results also suggest that MA_ActF_ during CPB may predict the fibrinogen concentration after CPB, and will thus enable the early and appropriate preparation for subsequent hemostatic management.

Tamura et al. [24] conducted a pilot study of the relationship between MA_ActF_ and plasma fibrinogen concentration during CPB in 17 patients who were undergoing cardiovascular surgery and calculated a Pearson’s correlation coefficient for the relationship between MA_ActF_ and plasma fibrinogen of 0.91. However, the correlation was calculated using multiple samples from the same patients measured at different times, and the sample size was too small to establish the reliability of the measurement, which may have led to an apparent overestimation of the correlation. We therefore performed an observational study based on the calculated required sample size, and we validated the usefulness of MA_ActF_ for the estimation of plasma fibrinogen concentration during CPB (*R* = 0.84). The results indicated that MA_ActF_ was strongly correlated with plasma fibrinogen concentration, even in highly heparinized blood samples. Because some transfusion strategies have been proposed in rotational thromboelastometry (ROTEM, TEM International GmbH, Munich, Germany) based on results showing correlations with fibrinogen concentration [14, 15], a coagulation management strategy can be developed for TEG6s, which has been proposed for use in ROTEM assays during CPB [14, 20, 21]. Our results serve as a reference for MA_ActF_ to intervene in the management of blood coagulation with TEG6s.

The ActF test measures blood viscoelasticity by evaluating fibrin network formation, triggered by reptilase and factor XIII. Because fibrin formation occurs in the absence of thrombin, it can be measured in highly heparinized blood, and we demonstrated a close relationship between MA_ActF_ and plasma fibrinogen concentration. In addition, we compared the outputs with the results of the Global Hemostasis assay using samples drawn after CPB and found a strong correlation between MA_ActF_ and MA_CFF_. Interestingly, in the same samples drawn after CPB, MA_ActF_ was always lower than MA_CFF_, and the mean difference between these two measures was 7.3 mm. However, there was no correlation between this difference and plasma fibrinogen concentration. The difference may be explained by structural differences in the networks formed in response to thrombin and reptilase. Thrombin cleaves fibrinopeptides A and B from the Aα-chain and Bβ-chain of fibrinogen, respectively, and the cleaved Aα-chain and Bβ-chain sites of fibrinogen then bind to the γ-domain of another molecule of fibrinogen, resulting in fibrin polymerization [28]. In addition, factor XIII activated by thrombin causes cross-linking of fibrin to form a strong fibrin mesh [29]. In contrast, reptilase only cleaves fibrinopeptide A from fibrinogen and does not cleave fibrinopeptide B [30] and the resulting fibrin polymerization and clot strength are therefore weak, despite the addition of activated factor XIII in the ActF measurement system. Considering these difference, different cutoff values must be set for MA_ActF_ and MA_CFF_. During cardiac surgery, the correlation coefficient for the relationship between TEG6s Global Hemostasis MA_CFF_ and plasma fibrinogen concentration before or after CPB has been reported to be 0.78 [23], which is similar to the strength of the correlation between MA_ActF_ and plasma fibrinogen concentration in the present study.

Fibrinogen is the most rapidly depleted clotting factor during massive hemorrhage; when its concentration falls below 100 mg/mL, clot formation does not occur [24]. According to recent guidelines, treatment should be started at a fibrinogen concentration of 150 mg/mL with bleeding [5]. Therefore, the estimation of fibrinogen concentrations during CPB may allow early intervention in patients who exhibit bleeding after CPB. Fibrinogen replacement therapy should be considered if the fibrinogen concentration is < 150 mg/dL with bleeding [5]. Furthermore, a fibrinogen concentration of < 200 mg/dL after CPB has been shown to increase the number of patients receiving large-volume red blood cell transfusions; a fibrinogen concentration of 200 mg/dL is necessary for sufficient recovery of fibrin polymerization according to thromboelastometry findings [10, 11]. Therefore, the cutoff values for detection of hypofibrinogenemia were defined as 150 and 200 mg/dL in the present study; we examined whether hypofibrinogenemia could be predicted by ActF. The cutoff value of MA_ActF_ for the measurement of a fibrinogen concentration of 150 mg/dL during CPB was 4.5 mm, and that for the measurement of a concentration of 200 mg/dL was 10.5 mm. Therefore, Platelet Mapping could be used to identify fibrinogen concentrations of 150 mg/dL or 200 mg/dL, which would be a trigger for the transfusion of FFP or other blood products to correct fibrinogen concentrations and maintain hemostasis following CPB in patients who are bleeding.

Bleeding due to coagulopathy in cardiac surgery is multifactorial [1–3]. Platelet Mapping was originally used as an assay to evaluate platelet inhibition and aggregation by antiplatelet drugs, such as ADP receptor antagonists and aspirin. In the field of cardiovascular surgery, preoperative Platelet Mapping measurements have been shown to be useful in predicting postoperative bleeding and transfusion strategies [31, 32].

The HKH test of the Platelet Mapping assay measures the clot strength associated with thrombin-mediated fibrin formation and platelets using reagents that contain kaolin and heparinase. In this context, the HKH test is designed using the same method as the CKH test of the Global Hemostasis assay; platelet count reportedly correlates with the MA yielded by the CK in the TEG6s Global Hemostasis assay [23, 33, 34]. Thus, HKH can theoretically reflect the viscoelasticity-induced platelet aggregation in a manner similar to the CKH test. The data from this study suggest that the MA yielded by the HKH test findings may be correlated with platelet count (Online Resource 1). Therefore, Platelet Mapping may serve as a more comprehensive viscoelastic coagulation assay. However, further studies are needed to investigate this potential application.

The study had several limitations. First, it was conducted in patients during and after CPB, and the results therefore cannot be extrapolated to patients who undergo surgery without CPB. Because Platelet Mapping ActF is based on the nonphysiological fibrin polymerization by reptilase, the Global Hemostasis CFF test is based on fibrin network formation induced by thrombin, while the Global Hemostasis assay evaluates residual heparin and whole blood coagulation; therefore, the Global Hemostasis assay is recommended for evaluation of blood coagulation after CPB. for patients who do not undergo high-dose heparinization. Second, the transfusion of FFP during CPB resulted in a narrow range of plasma fibrinogen concentrations in the study, but more profound hypofibrinogenemia (e.g., < 100 mg/dL) might limit the polymerization of fibrin in response to reptilase, leading to low MA_ActF_ values. Consistent with this hypothesis, Tamura et al. reported that MA_ActF_ was 0 mm in samples with fibrinogen concentrations of ≤ 100 mg/dL [24]. Therefore, we believe that this will have had a limited effect on our findings. Third, this was an observational study, and was not designed to determine if clinical decisions based on the Platelet Mapping assay results during CPB resulted in a reduction of bleeding or amount of transfusion. However, our results will provide a theoretical background for further prospective randomized trials to validate the usefulness of measuring MA_ActF_ during CPB. Finally, because this was a single-center study, further multicenter studies are needed to determine whether the findings can be generalized to other populations.

In conclusion, TEG6s Platelet Mapping ActF MA correlates with plasma fibrinogen concentration in highly heparinized blood samples collected from patients undergoing CPB and thus provides a highly accurate means of identifying fibrinogen concentrations that are ≤ 150 mg/dL. These results suggest that the Platelet Mapping assay would be useful for estimating fibrinogen concentrations during CPB. Further studies are needed to confirm the clinical efficacy of this assay.

## Supplementary Information

Below is the link to the electronic supplementary material.

Supplementary file1 (PDF 73 kb)
