# Supplementary material for: TEG6s Platelet Mapping assay for the estimation of plasma fibrinogen concentration during cardiovascular surgery: a single-center prospective observational study
Source: J Anesth. 2021 Oct 13;36(1):79–88. doi: 10.1007/s00540-021-03009-4 (PMC8807459; doi:10.1007/s00540-021-03009-4)
Supplement: Supplementary file 1 — Supplementary file1 (PDF 73 kb) [file 540_2021_3009_MOESM1_ESM.pdf]

Online Resource 1

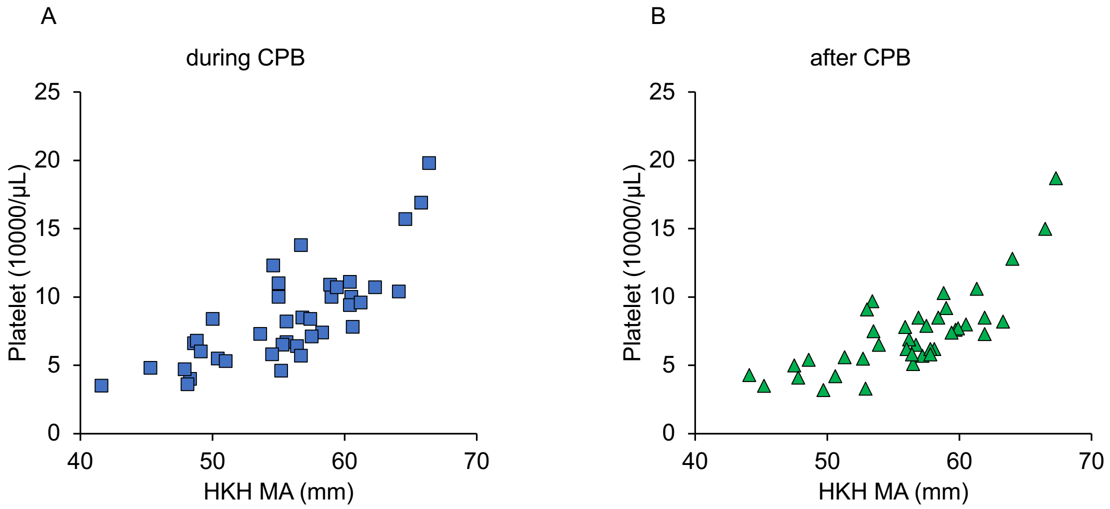

Relationship between the MA yielded by PlateletMapping HKH tests and the platelet count

(A) During CPB (*R*=0.77, 95% CI 0.61–0.87; *P*<0.001). (B) After CPB (*R*=0.76, 95% CI 0.59–0.87; *P*<0.001). During CPB: samples were drawn after the declamping of the aorta during CPB; after CPB: after heparin reversal using protamine. HKH, kaolin and heparinase activation test; CI, confidence interval; CPB, cardiopulmonary bypass; MA, maximum amplitude; *R*, Pearson's correlation coefficient.
